# Construction of helper plasmid-mediated dual-display phage for autoantibody screening in serum

**DOI:** 10.1007/s00253-014-5713-8

**Published:** 2014-04-25

**Authors:** Kaushik Rajaram, Veronique Vermeeren, Klaartje Somers, Veerle Somers, Luc Michiels

**Affiliations:** 1 Biomedical Research Institute, Hasselt University, Martelarenlaan 42, 3500 Hasselt, Belgium; 2 Complix NV, BioVille, Agoralaan building A-bis, 3590 Diepenbeek, Belgium

**Keywords:** Phage ELISA, Streptavidin-binding protein, Helper plasmid, Rheumatoid arthritis, Autoantibody, Dual display

## Abstract

M13 filamentous bacteriophage has been used in displaying disease-specific antibodies, biomarkers, and peptides. One of the major drawbacks of using phage in diagnostic assays is the aspecific adsorption of proteins leading to a high background signal and decreasing sensitivity. To deal with this, we developed a genetically pure, exchangeable dual-display phage system in which biomarkers and streptavidin-binding protein (SBP) are displayed at opposite ends of the phage. This approach allows for sample purification, using streptavidin-coated magnetic beads resulting in a higher sensitivity of signal detection assays. Our dual-display cassette system approach also allows for easy exchange of both the anchor protein (SBP) and the displayed biomarker. The presented principle is applied for the detection of antibody reactivity against UH-RA.21 which is a good candidate biomarker for rheumatoid arthritis (RA). The applicability of dual-display phage preparation using a helper plasmid system is demonstrated, and its increased sensitivity in phage ELISA assays using patient serum samples is shown.

## Introduction

Phage display (PD) has taken its stand in the era of molecular biology in the last two decades by displaying a large number of proteins, antibodies, amino acids, etc. on the surface of phage (Reynolds et al., 2011; Soendergaard et al., 2011). The non-lytic M13 filamentous phage is a single-stranded (ss) DNA virus that infects a number of gram-negative bacteria. A phage particle consists of a long cylindrical protein structure, 800–900 nm in length and 6.5 nm in diameter. It has one major coat protein, pVIII, that surrounds the entire phage body with 2,700 copies, and each end has two minor coat proteins (pVII, pIX and pVI, pIII), each present in 3–5 copies. All these coat proteins contribute to the stability of the phage (Sidhu, 2001; Sidhu, 2000; Arap 2005). The phage genome carries the genes for these five coat proteins and the genes for the proteins involved in phage replication. PD involves the fusion of a foreign peptide or protein with a minor or major coat protein of the phage through genetic recombination of the phage genome with the complementary deoxyribonucleic (cDNA) of the insert, resulting in the phage particle displaying the peptide or protein fused to the coat protein, and also possessing the gene of the protein insert, providing a direct link between the phenotype and the genotype (Dover et al. 2009).

Between the two popular vector systems, using a phagemid vector system to produce functional phage particles is better than using a phage vector, showing a good display level of the fusion protein, and being genetically more stable in displaying larger proteins (Sidhu, 2001). However, a phagemid vector carries only the gene for the coat protein to which the cDNA of the protein of interest is fused. So, after transformation of the bacterial host with phagemid, it needs a coinfection from a helper phage to provide the native genes for all the other structural proteins to make a complete and functional phage. The genome of the helper phage lacks a packaging signal, so the genome of the phagemid vector will preferentially get packaged into the new phage particles, retaining the link between the phenotype and the genotype of the phage (Qi et al. 2012; Bratkovic, 2009). Chasteen and coworkers proposed a different vector system as an alternative to the use of helper phage, making use of helper plasmids, providing multivalent display and genetically pure phage (Chasteen et al. 2006). As with the helper plasmid/phagemid vector system, phage produced by this method contains only the genome of the phagemids. Although a dual-display system in T4 phage using bipartite SOC-HOC system and lambda phage is attractive because this allows for a higher copy number and there is no need for secretion through the cell membrane; most of the biomarkers (C DNA libraries) have been developed from M13-based phagemid system (Ren and Black 1998; Ren et al. 1996; Pavoni et al. 2013). Moreover, the RA biomarkers that are addressed in this work have also been selected using the M13 phage display system. Therefore, we aim at the development of a new strategy to improve the sensitivity of already available biomarkers and possibly taking into sensing platform.

Protein aggregation and lack of protein transfer to the bacterial periplasm are often observed and result in the failure of phage display. This problem can be addressed by the introduction of a leader peptide or signaling peptide into the fusion protein. However, the correct folding can be compromised due to the secretion of the phage from the host (Velappan et al. 2010). Therefore, coat proteins pVIII and pIII are commonly used for protein display because those genes contain N-terminal periplasm-directing signal sequences. By introducing such a signal sequence between the N-terminus of pVII or pIX and the cDNA of the protein of interest, it becomes possible to develop a stable display system using those coat proteins as well (Georgieva and Konthur 2012; Loset and Sandlie 2012). There is a persisting demand for suitable disease-related biomarkers as indicators for diseases, like proteins, peptides, metabolites, and antibodies (Vithayathil et al. 2011). The capability to display disease-specific proteins on a phage surface could be a big advantage in the field of diagnostics. Phage can be directly used as a probe when it is modified to display disease-related antigens on its surface (Kierny et al. 2012). Also, problems associated with synthesizing small molecules can be overcome by PD (Dudak et al. 2011). This would involve the phage in the development of diagnostic biosensors, functioning as a receptor, attached to an electrode or transducer surface. Increasing attention has been drawn towards the use of phage as a receptor molecule in different applications, due to its inherent stability and resistance to denaturation in unfavorable conditions, unlike DNA and antibodies. It can sustain higher temperatures of up to 70 °C and pH variations from 2.5 to 12 (Arap 2005; Mao et al. 2009).

Phage production is also cost-effective, in contrast to the production of monoclonal antibodies. They can be produced in sufficient numbers by just infecting a bacterial host. Phage-based assays are mostly performed in an ELISA format; however, if complex substances such as serum are used in detecting targets, this approach lacks sensitivity due to aspecific binding of interfering serum components increasing the background signal. The same problem can be expected in the abovementioned biosensor setups. Therefore, sample preparation should also be taken into account when setting up such assays.

The design of a dual-display system, where phage displays both a biomarker and a capturing protein, allows for such a sample preparation. In the presented approach, the dual-display phage will display a biomarker fused to pVI at one end and an anchoring streptavidin-binding protein (SBP) fused to pVII at the other end of the phage. SBP will be binding to streptavidin-coated magnetic beads allowing a magnetic capturing of the phage. As a proof of principle, a candidate biomarker for rheumatoid arthritis (RA) UH-RA.21 (Somers et al. 2011; Somers et al. 2005) is used to screen for the presence of autoantibody reactivity in the serum of RA patients in comparison to basic ELISA procedures.

## Materials and methods

### Vectors and minigene

M13cp helper plasmid with chloramphenicol (Chlr) resistance in a DH5αF′ bacterial host was kindly provided by ARM Bradbury’s lab (Las Alamos, USA) (Chasteen et al. 2006). pspB RA21 phagemids and pspB empty phagemids with Amp resistance in TG1 bacterial hosts (Somers et al. 2009) and serum samples with different levels of RA autoantibodies (highly positive, moderately positive, borderline positive) as well as healthy control serum samples were produced in-house. Helper phage with kanamycin (Kan) resistance was purchased from GE healthcare (Diegem, Belgium). Ampicillin-resistant IDTB vector containing SBP minigene gVII-SBP was purchased from IDT (Leuven, Belgium). For plasmid and phagemid purification from host bacterial cells, Qiagen midi prep kit from Qiagen (Antwerp, Belgium).

### gVII-SBP minigene cloning into the genome of the M13cp helper plasmid

Restriction enzymes *Sna*B I and *Bsp* 1407I (TaKaRa Bio Inc (Japan)) were used to replace the gVII of the helper plasmid with the SBP minigene. Five microliters of ligation product was then transformed into a freshly prepared chemically competent DH5αF′ bacterial strain, by subjecting the bacteria to a heat shock at 42 °C for 30 s, after which they were plated on LB agar (Invitrogen, Ghent, Belgium) plates with 15 μg/ml of the antibiotic Chlr and incubated at 37 °C overnight. A negative control was also prepared where the DH5αF′ bacterial strain was transformed with unmodified M13cp. DH5αF′ cells already containing the Chlr-resistant M13cpSBP or M13cp helper plasmid were prepared to be competent and cotransformed with Amp-resistant phagemid pspB, either empty (pspB) or bearing the cDNA of the autoantigenic target RA21 fused to its pVI gene (pspB RA21), in the same way as with the helper plasmid. The newly cotransformed DH5αF′ colonies were plated in 2×YT media (BD (Erembodegem, Belgium)) containing 15 μg/ml of Chlr and 100 μg/ml of Amp.

### Colony PCR and sequencing

Positive colonies grown in the antibiotic selective plates were used in colony PCR with the forward primer 5′-AAT GTT GTT CCG TTA GTT CG-3′ and reverse primer 5′-CCA TTA AAC GGG TAA AAT AC-3′ (Eurogentec (Seraing, Belgium)) for helper plasmid transformed with SBP minigene, and the primer sets gVI forward primer 5′-TTA CCC TCT GAC TTT GTT CA-3′ and pUC 19 reverse primer 5′-CGC CAG GGT TTT CCC AGT CAC GAC-3′ were used for phagemids. The thermocycling conditions included an initial denaturation at 95 °C for 7 min, followed by 30 cycles comprising of a 30-s denaturation step at 95 °C, a 30-s annealing step at 55 °C, and a 4-min elongation step at 72 °C, and one final elongation step carried out at 72 °C for 10 min. These PCR products have been used without any further purification in sequencing with the same forward primers as mentioned above by using ABI PRISM Genetic Analyzer 310 (Applied Biosystems (Warrington, UK)). The sequences were analyzed using Chromas software version 2.13 and DNAMAN version 7.0.

### Phage production

Dual SBP-RA21 and single SBP or UH-RA.21 display phage were produced from the double transformed DH5αF′ bacterial cells. A single colony from the plate was picked and grown until they attained an exponential growth rate in 2×YT medium containing 15 μg/ml of Chlr and 100 μg/ml of Amp. Then, 4 ml of exponentially growing cells was transferred into 50 ml of fresh 2×YT broth medium with both antibiotics. Subsequently, they were incubated in a shaking incubator at 200 rpm for 16 to 18 h at 31 °C. Afterwards, all the bacterial cells were pelletized by centrifuging at 4,000 rpm, and then, the supernatant was added with 20 % 6000 MW PEG (Merck (Darmstadt, Germany)) in 2.5 M NaCl and kept on ice for 1 h. They were centrifuged again at 4,000 rpm for 20 min. The obtained white phage pellets were washed with 1 × phosphate-buffered saline (PBS) until all the remaining bacterial cells were removed.

In addition, to produce positive control phage, Std21, TG1 bacterial cells bearing the phagemid pspB RA21 were grown up to the exponential phase, and 10 ml of exponentially grown cells was added with 5 μl of M13KO7 helper phage. The helper phage was allowed to infect the TG1 cells for 30 min in a 37 °C water bath, and the solution was then incubated in a shaking incubator for 10 min at 100 rpm while keeping the same temperature. These infected cells were added to fresh 2×YT medium containing 100 μg/ml Amp and 40 μg/ml Kan and grown overnight at 30 °C. After the phage production, the amount of phage was tittered by using PR phage titration kit (Progen Biotechnik GmbH (Germany)). The absorbance values of phage samples were extrapolated with the standard graph made from the absorbance values of the known phage standards from the kit.

### Phage ELISA

In order to check the SBP display, ELISA microtiter plates (Greiner Bio-One BVBA, Wemmel, Belgium) were coated overnight with 5 μg/ml anti-pVIII antibodies. In the finding of RA21 display and dual expression at the same time, ELISA microtiter plates were coated overnight with 10 μg/ml anti-human IgG antibodies (Dako, Denmark) as mentioned in Table 1. Afterwards, the plates were washed twice with 1 × PBS. Then, the wells were blocked with 5 % Marvel skim milk powder (Chivers, Dublin Ireland) in 1 × PBS (MPBS) for 2 h at 37 °C while shaking. The plates were washed three times with 1 × PBS containing 0.1 % of between 20 (PBST) and once with 1 × PBS. Simultaneously, all the phage samples were diluted to 10^12^ colony-forming units (CFU)/ml in 5 % MPBS.

**Table 1:** Components of phage ELISA

Target assay	Coating antibody	Sample	Detection antibody/entity
SBP	Anti-M13	Phage	Streptavidin HRP
UH-RA.21	Anti-human IgG	Pre-incubated phage and serum	Anti-M13 HRP
Dual display	Anti-human IgG	Pre-incubated phage serum and streptavidin bead	Anti-M13 HRP

This table comprises the different assays and their coating antibodies, nature of sample, and detection antibodies used

In order to confirm the RA21 and dual expression, 100 μl of 1 × 10^12^ CFU/ml of phage in 5 % MPBS was first pre-incubated with 100 μl of 100-fold in 5 % MPBS diluted RA patient’s serum with different levels of anti-RA21 autoantibodies (highly positive, moderately positive, borderline positive, and negative (healthy control)) at 37 °C for 30 min under static conditions and for 30 min while shaking at 100 rpm. For the evaluation of dual expression, 1 μl of 10 μg/μl (3–6 × 10^6^) streptavidin-coated magnetic beads (Invitrogen, Ghent, Belgium) was added to the phage-serum mixture and incubated again at 37 °C for 30 min while shaking. The phage-serum-bead complexes were captured with a magnetic field, and washed twice with 5 % MPBS to remove unbound phage or remaining serum, and resuspended in 5 % MPBS.

For SBP display confirmation, 100 μl of pure 10^12^ CFU/ml phage was added to the wells. For confirmation of RA21 expression, 100 μl of the pre-incubated phage and serum samples were added to the wells. For dual expression study, 100 μl of the pre-incubated phage/serum/bead complex was added to the wells. They were incubated for 1 h under static conditions and for 30 min while shaking at 37 °C. After washing the titer plate as described above, 0.83 μg/ml of a streptavidin-horseradish peroxidase (HRP) conjugate was added for the detection of SBP, and incubated for 1 h at RT while shaking. For the studies of RA21 and dual expression, polyclonal anti-M13 antibodies conjugated with HRP from the phage titration kit were added as secondary antibodies and incubated for 1 h at RT while shaking. After washing, ready-to-use TMB and H_2_O_2_ (Thermo Scientific (Erembodegem, Belgium) were used as a substrate solution for HRP, inducing a color reaction, and 2 M H_2_SO_4_ was used as a stop solution. The plate was read at a wavelength of 450 nm to get the absorbance values.

### Dot blotting

Different 5 μl spots with 10^13^ CFU/ml of phage were applied to a WhatMan nitrocellulose membrane filter paper (WhatMan GmbH, Germany). The spots were allowed to dry for 15 min, and then, the surface was blocked with 5 % MPBS for 15 min. After rinsing two times in 1 × PBS, the nitrocellulose filter paper was treated with 0.83 μg/ml of streptavidin-HRP for 1 h at RT while shaking. The paper was washed three times with 1 × PBS containing 0.5 % Triton™ X-100 and twice with 1 × PBS. Finally, fresh DAB and H_2_O_2_ (Thermo Scientific (Erembodegem, Belgium) solution was added to develop the colored spots.

### Statistical analysis

Statistical analysis was performed using a trial version of GraphPad InStat version 3.1. Non-parametric ANOVA (Kruskall-Wallis testing) and Dunn’s multiple comparison tests were used to compare the reactivity against the serum samples with the different levels of anti-UH-RA.21 positivity. The levels of statistical significance were as follows: a *P* value of <0.05 (*), a *P* value of <0.01 (**), and a *P* value of <0.001 (***).

## Results

### Construction of the M13cpSBP helper plasmid

**Fig. 1:**
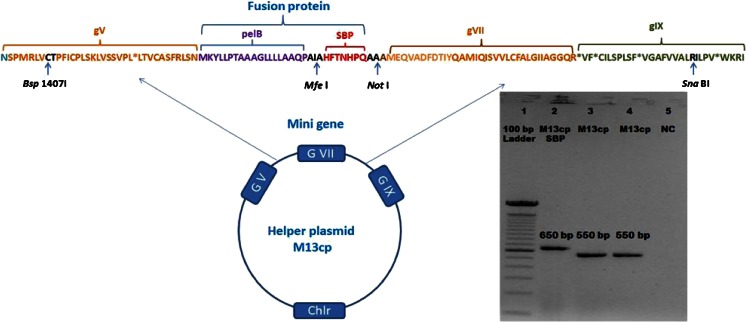
Construction of the helper plasmid M13cpSBP. A minigene was constructed in which PelB (*purple*) and SBP (*red*) coding sequences are inserted upstream of the gVII gene of wild-type M13cp phage DNA. The SBP sequence is flanked by the restriction sites *Not I* and *Mfe I*. The entire gVII-SBP minigene is located between gV and gIX and is contained between the restriction sites *Sna*BI and *Bsp*1407I. Figure 1 insert—A 1 % agarose gel demonstrates the successful insertion of SBP and PelB into gVII of M13cp. *Lane 1*, 100 bp ladder; *lane 2*, recombinant M13cpSBP construct; *lanes 3* and *4*, wild-type M13cp; and *lane 5*, negative control. Sequencing of the M13cpSBP confirmed the in-frame insertion of the SBP and pelB sequences as shown in the presented sequence

Displaying proteins, such as SBP in this case, on pVII is feasible but only if the cDNA of the insert is placed between the gVII start codon and a signaling or a leader peptide (Loset and Sandlie 2012; Kierny et al. 2012). Figure 1 shows the design and construction of the helper plasmid M13cpSBP. The most common signaling peptide PelB is used to ensure the correct folding of SBP and its transport to the bacterial membrane, and eventually to the phage surface (Kwasnikowski et al. 2005). A predesigned SBP minigene consisting of the cDNA of the signaling peptide sequence PelB and the cDNA of SBP is inserted upstream of the start codon of gVII of the helper plasmid M13cp (Fig 1). This SBP minigene was designed between two restriction sites *Sna*B I and *Bsp*1407I which allows easy insertion into the gVII of the wild-type helper plasmid M13cp. In addition to this, the coding sequence of the anchor peptide SBP itself is flanked with two different restriction sites *Not* I and *Mfe* I, which makes SBP easily interchangeable with another type of anchoring peptide. The resulting SBP modified helper plasmid was termed M13cpSBP, and it was transformed into a DH5αF′ bacterial host. As a negative control, unmodified helper plasmid M13cp was also transformed.

After transformation, successful insertion of the SBP minigene into the helper plasmid M13cp was confirmed by colony PCR and sequencing. Figure 1 insert shows the ∼100 bp longer fragment of M13cpSBP (lane 2) as compared to unmodified M13cp (lanes 3–4); the sequence of the resulting construct shown in Fig. 1 was confirmed by sequencing analysis.

### M13cpSBP and wild-type phage production

**Fig. 2:**
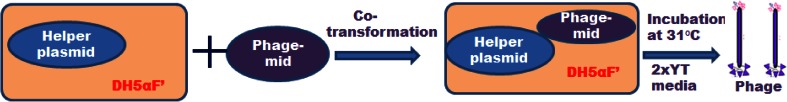
Graphical representation of the cotransformation and resulting phage preparation DH5αF′ host bacteria, containing the M13cpSBP or M13cp helper plasmid, were cotransformed with UH-RA.21 autoantigenic target bearing or empty phagemid. Bacteria containing both plasmids were grown in 2×YT medium to produce phage particles

**Fig. 3:**
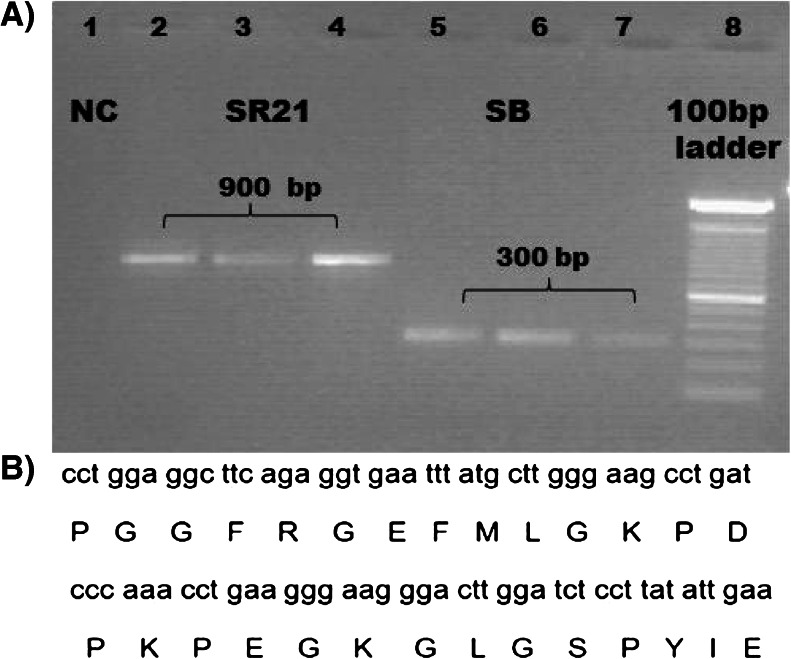
Cotransformation of DH5αF′ cells with pspB phagemids. **a** 1 % agarose gel demonstrating the difference of ∼600 nucleotides, corresponding to the 28 amino acids of the UH-RA.21 marker and the linker sequences between the dual-display phage SR21 (lanes 2, 3, and 4) and the single-display phage SB (5, 6, and 7). Lane 1 shows the result of the colony PCR for RA21 performed on DH5αF’ bacteria cotransformed with native pspB. **b** Sequence analysis confirms the in-frame sequences of RA21 and its translation product is shown

In order to produce phage particles, a helper plasmid always needs support from a phagemid, since a helper plasmid lacks a packaging mechanism. So, Dh5αF′ cells bearing either modified M13cpSBP or unmodified M13cp helper plasmid were cotransformed with pspB carrying UH-RA.21 and pspB phagemids (Somers et al. 2011) without insert (empty phagemid) and plated on 2×YT plates containing chloramphenicol (Chlr) and Amp. These antibiotics are used to select bacteria containing both helper plasmid (with and without SBP) and phagemid (with and without UH-RA.21). A schematic overview of the cotransformation and phage production procedure is shown in Fig. 2. Chlr- and Amp-resistant cells were subjected to colony PCR, and the resulting products were analyzed by gel electrophoresis (Fig. 3a). The colony PCR products show the additional 600 bp in the SR21 phage (lanes 2–4) corresponding to the UH-RA.21 marker, as compared to the SB phage (lanes 5–7). Sequencing analysis (Fig. 3b) confirmed the sequences of RA21 (Somers et al. 2011). One of the confirmed positive SR21 colonies containing the two targets SBP and RA21 was selected for phage production in 2×YT medium. This helper plasmid-mediated phage production leads to genetically pure phage because the M13cp helper plasmid does not have any packaging signal and delivers only proteins. The phagemid genome carrying the gVI-RA21 cDNA is preferably packed into each phage (Chasteen et al. 2006). Titer values of all the phage particles produced range from 1 × 10^13^ to 5.5 × 10^13^ CFU/ml.

In this way, four different phage samples were prepared using this approach as is summarized in Table. 2. The standard positive control phage (Std21), which has been used previously in conventional phage ELISA protocols (Somers et al. 2011), is prepared differently by infecting a bacterial host containing UH-RA.21 in pspB phagemids with helper phage M13, results in phage displaying RA21 at pVI of the phage and contains other wild-type proteins from helper phage M13.

**Table 2:** Types of phage produced and protein displayed and its titer

Name of the phage	Displayed protein	Titer in CFU in 10^13^
SR21	SBP and RA21	2.4
SB	SBP	2.1
CR21	RA21	1.7
CB	–	3.1
Std21	RA21	5.3

The first four phages are the different types of phage produced using phagemid and helper plasmid system. Std21 is the conventional phage prepared and used as a positive control in phage ELISA

### Evaluation of the expression of SBP in pVII display phage

Theoretically, phage SR21 and SB should display SBP on all five copies of the coat protein pVII of the phage particle, due to the lack of native gVII in the pspB phagemid. Moreover, SBP can be displayed at a higher local concentration due to the small size of pVII and the tightly packed system (Kwasnikowski et al. 2005).

**Fig. 4:**
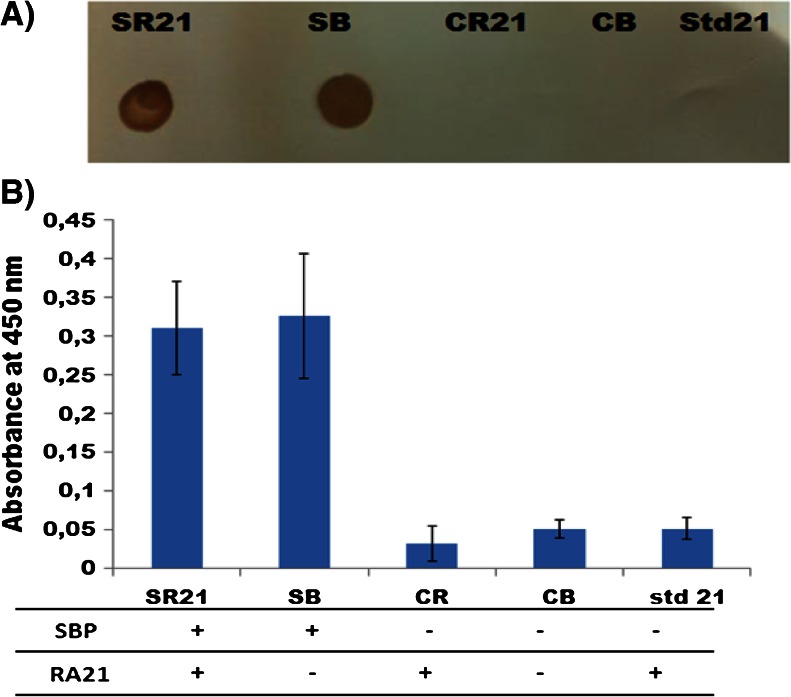
Expression of SBP in pVII in displaying phage. The expression of SBP was analyzed in four types of phage (SR21, SB, CR, and CB) produced through the helper plasmid/phagemid system and compared to phage (Std21) produced through the helper phage/phagemid system. **a** *Dot blot* shows the four types of phage and the Std21 phage on a nitrocellulose paper, using a SBP-dependent color reaction. **b** Phage ELISA on anti-pVIII-coated plates, treated with the four types of phage and the Std21 phage, and detected with a streptavidin-conjugated HRP and a TMB-dependent color reaction

This is evident from the immediate strong color formation in a dot blotting experiment, as shown in Fig. 4a. Among the five different phage preparations, the dual-display phage SR21 and one of the single display phage, SB, are positive, while the other phage types remain negative. This was confirmed in a phage ELISA format, as shown in Fig. 4b, in which SR21 and SB show a six-fold absorbance compared to the negative phage CR21 and CB. As a control in both experiments, phage produced from the cotransformation of DH5αF′ with SBP-negative helper phage and pspB carrying RA21, referred to as Std21, was also checked for SBP expression, and was found to be negative for SBP expression.

### Screening of RA patient sera using UH-RA.21 displaying phage

**Fig. 5:**
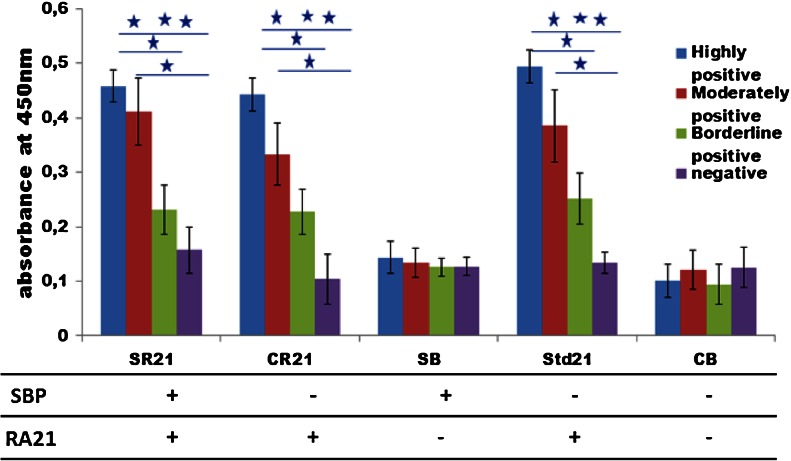
Screening RA patient sera for the presence of autoantibodies with RA21 displaying phage preparations. All four types of phage (SR21, CR21, SB, and CB) were produced through the helper plasmid/phagemid system as compared to phage (Std21) produced through the helper phage/phagemid system by phage ELISA. The four types of phage and the Std21 phage were pre-incubated with serum that was highly positive (*blue*), moderately positive (*red*), borderline positive (*green*), and negative (*violet*) for antibody reacting against UH-RA.21 and captured onto anti-human IgG-coated ELISA plates. SR21, CR21, and Std21 were able to discriminate highly and moderately positive serum from negative serum by showing an increased absorbance at 450 nm. However, discrimination between borderline positive and negative serum was not possible. The statistical comparison of the phage ELISA results among the sera with different levels of autoantibody positivity has been given in the form of stars (*/**/***) which corresponds to the approximate *P* values of *P* < 0.05 (significant), *P* < 0.01(good significant), and *P* < 0.001 (excellent significant) respectively. This experiment was carried out three times, with each condition in duplicate

As a proof of concept, we have used one of the most promising RA autoantigenic targets RA21 which was displayed on pVI of the filamentous phage M13 (Somers et al. 2009; Somers et al. 2011). The expression of RA21 was evaluated in phage ELISA using the sera from RA patients who were classified according to the amount of autoantibodies present in their serum (either highly positive, moderately positive, or borderline positive), and serum from healthy controls who are negative for anti-RA21 autoantibodies. These sera were allowed to react with the five types of phage displaying UH-RA.21 (SR21, CR21, Std21) or not displaying (SB, CB) UH-RA.21. Autoantibodies and phage complexes were captured onto anti-human IgG-coated ELISA plates. Anti-M13 (anti-pVIII) polyclonal antibodies conjugated with HRP were used as detection antibodies in a sandwich ELISA protocol. Figure 5 shows the ELISA results of the Std21 phage preparations, which is used in a standard phage ELISA protocol to the single (CR21) and the dual-display phage (SR21). Both CR21 and SR21 show high levels of absorbance in the phage ELISA when treated with serum from highly, moderately, and borderline positive patients for anti-RA21 autoantibodies. This is comparable to the standard ELISA procedure using Std21 phage. All three phage types (SR21, CR21, and Std21), however, were not able to distinguish the borderline positive sera from the negative control sera significantly. Nevertheless, our dual-display phage system did not interrupt the display of UH-RA.21 and performs well in phage ELISA, comparable to Std21 phage used up to now. This confirms the expression of the UH-RA.21 marker in our phage preparations.

### Screening RA patients sera for UH-RA.21 autoantibodies after sample purification based on dual-display phage

**Fig. 6:**
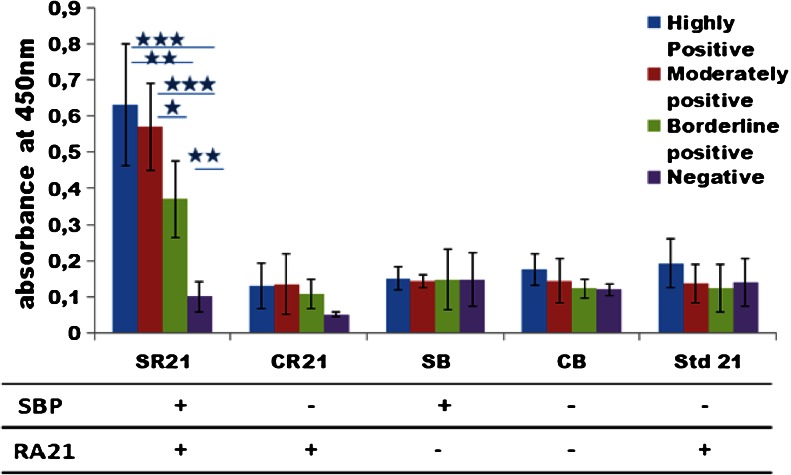
Screening RA patients sera for UH-RA.21 autoantibodies after sample purification based on dual-display phage: All four types of phage (SR21, CR21, SB, and CB) produced through the helper plasmid/phagemid system, as compared to phage produced through the helper phage (Std21) system by phage ELISA. The four types of phage and the Std21 phage were pre-incubated with serum that was highly positive (*blue*), moderately positive (*red*), borderline positive (*green*), and negative (*violet*) for anti-UH-RA.21, and isolated with streptavidin-coated magnetic beads. The resulting complexes were captured onto anti-human IgG-coated ELISA plates. The statistical comparison of the phage ELISA results among the sera with different levels of autoantibody positivity is shown in which */**/*** which corresponds to approximate *P* values of *P* < 0.05 (significant), *P* < 0.01(good significant), and *P* < 0.001 (excellent significant), respectively. These experiments were carried out three times, with each condition in duplicate

The five types of phage were again pre-incubated with the sera from RA patients containing different levels of anti-UH-RA.21 antibody positive (highly positive, moderately positive, and borderline positive) and with anti-RA21-negative sera from healthy controls, to allow recognition of the displayed RA21. The phages with and without bound serum autoantibodies were subsequently isolated with streptavidin-coated magnetic beads, binding to the displayed SBP. These complexes of phage and serum autoantibodies captured on streptavidin-coated magnetic beads were then added to anti-human IgG-coated ELISA plates and detected with anti-M13 antibodies conjugated with HRP. All phages displaying SBP are bound to and isolated by the magnetic beads. However, of those, only the one displaying RA21 will bind to the microtiter plate through an anti-RA21/anti-human IgG complex, and generate a color reaction. Figure 6 shows very clearly that dual-display phage SR21 which is pre-incubated with the positive sera can specifically bind to the autoantibodies in the serum with its pVI-displayed UH-RA.21 autoantigenic target and also to the streptavidin-coated magnetic beads by its pVII-displayed SBP at the same time, and generate a positive ELISA result. This dual-display system allows magnetic capture of phage complex and allows specific selection of dual-display phage in assays, that gives higher sensitivity. Moreover, the absorbance values for the borderline positive sera still increased significantly as compared to the negative serum. In other words, using a dual-display phage system, a higher sensitivity is reached in RA diagnosis, allows specific detection of an additional group of patients, displaying only low amounts of anti-UH-RA.21 autoantibodies. Since these patients could still be in an early stage of the disease, this could have tremendous impact on prognosis.

## Discussion

In a previous work, Somers et al. (2009) and Somers et al. (2011) have identified a panel of 14 autoantigenic targets for RA in a pVI-displayed cDNA library using SAS technology. One of these 14 biomarkers, UH-RA.21 shows a high sensitivity to detect early RA patients who are rheumatoid factor-negative (RF−) and anti-cyclic citrullinated peptide (ACCP−) negative (Somers et al. 2009). This biomarker was chosen as a proof of principle target in this study. In order to increase the assay sensitivity, we introduced a short SBP peptide (HFNTHPQ) into the gene VII (gVII) of the helper plasmid, which contains a his-pro-glu (HPQ) motif that allows strong streptavidin binding (Dudak et al. 2011; Kwasnikowski et al. 2005; Chen et al. 2004). It is a very strong natural non-covalent coupling with a dissociation constant of ∼2.5 × 10^8^ M (Chen et al. 2004). In this report, we have demonstrated a novel approach to produce a dual-display phage, displaying a disease-related biomarker for screening at one end of the phage, and an anchor peptide for attachment to a sensing platform at the other end, for an easy, rapid, and cost-effective way of diagnosis. In order to achieve this, cotransformation of bacterial host cells with a phagemid vector providing the autoantigenic target UH-RA.21 and with a helper plasmid providing the anchoring peptide SBP was performed. This SBP was fused with a leader peptide pelB, at the pVII of helper plasmid M13cp. The resultant phage displayed the autoantigenic target UH-RA.21 at one end and SBP at the other end.

Four different types of phage displaying, both UH-RA.21 and SBP (SR21), either UH-RA.21 (CR21) or SBP (SB), and non-displaying (CB) were produced by cotransforming helper plasmids bearing bacterial cells with phagemid. And the conventional positive control phage Std21 is produced via helper phage, and phagemid system (Std21) was used to compare the efficiency of different RA21 displaying phage. The SBP end of a phage will allow capture of the phage in a well-oriented fashion on a streptavidin-functionalized sensor surface by forming strong affinity bonds or on magnetic beads allowing for the isolation of autoantibodies from the serum of a patient. At the same time, the other end of the phage is readily available to screen for the autoantibodies in the patient’s material.

Phage ELISA and dot blotting has been used in characterizing the display of SBP, and the results confirms the clear expression of SBP in both SR21 and SB phage from few-fold higher absorbance compared to the other phage samples in ELISA and immediate strong color formation in dot blotting. Phage ELISA was also used to characterize the displaying capacity of UH-RA.21 in three phage samples RA21, CR21, and positive control phage Std21. As in standard ELISA procedures, all show higher absorbance in phage ELISA with the patient’s serum samples although it is not significantly discriminating the borderline positive serum from negative control (healthy control).

However, using our dual-display approach sample purification process using streptavidin-coated magnetic beads can be used to isolate autoantibodies in the serum selectively by the phage displaying UH-RA.21 and SBP. In addition, it was shown that the optimal use of dual-display phage in the screening for anti-RA21 autoantibodies in patient sera is resulting in an increased assay sensitivity. Even patients that are borderline positive for the presence of autoantibodies, and that could not be discriminated from healthy controls using regular phage ELISA approaches, show a significant increase in absorbance, and can be clearly discriminated from healthy controls. Moreover, this system is designed as a cassette-setup to allow the exchange of SBP with any other anchoring peptide and UH-RA.21 with any other biomarker depending on varying needs. In the future, this dual-display phage can be used as a receptor attached to a sensing platform for rapid screening, making a label-free and highly specific diagnosis of RA and other diseases possible.
